# Aortic Dissection in a Young Woman: A Painless Presentation With Stroke-Like Symptoms and Horner’s Syndrome

**DOI:** 10.7759/cureus.85764

**Published:** 2025-06-11

**Authors:** Blake Edwards, Anita M Medepalli, Muhammad Khan

**Affiliations:** 1 Internal Medicine, Mercer University School of Medicine, Macon, USA; 2 Medical School, Mercer University School of Medicine, Macon, USA; 3 Pulmonology and Critical Care, Atrium Health Navicent The Medical Center, Macon, USA

**Keywords:** aortic dissection, carotid dissection, embolic stroke, horners syndrome, internal carotid artery occlusion, paraplegia, stroke

## Abstract

Aortic dissection is a rare cardiovascular emergency that can present atypically, complicating diagnosis and management. We report the case of a woman in her 30s with Stanford Type A aortic dissection, presenting with stroke-like symptoms and Horner’s syndrome without chest pain. Initial management focused on her neurological deficits, delaying the diagnosis. Bedside echocardiography and confirmatory imaging ultimately revealed an extensive dissection. Despite medical management, the patient succumbed to her condition. This case underscores the importance of recognizing atypical presentations of aortic dissection to enable timely diagnosis and improve outcomes.

## Introduction

Aortic dissection is a life-threatening cardiovascular emergency in which a tear in the inner layer of the aorta allows blood to flow between the layers of the aortic wall [1]. The clinical presentation is highly variable, often making diagnosis challenging. It is characterized by rapid onset, relentless progression, and high mortality rate. The estimated incidence is 2.5-3.5 cases per 100,000 person-years [2]. 

The hallmark clinical feature of aortic dissection is a sudden onset of chest pain radiating to the back; however, a painless aortic dissection can occur in 5-15% of cases [3]. Therefore, a diagnosis of aortic dissection can be elusive in a clinical presentation without the sudden onset of pain. 

This case report highlights an unusual presentation of an extensive Stanford Type A aortic dissection in a young female patient who presented with Horner’s syndrome and stroke-like symptoms without the classic feature of chest pain. It aims to contribute to the growing body of literature on painless aortic dissections and emphasize the critical importance of rapid recognition and intervention in these atypical cases.

## Case presentation

A woman in her late 30s presented to the emergency department (ED) with a four-hour history of confusion, agitation, a sensation of feeling overheated, and sudden left-sided weakness following recent methamphetamine use. Due to her acute confusional state, a reliable history could not be obtained. However, according to her friend and a relative, her medical, surgical, and family history was unremarkable. She had no known allergies and no recent travel or sick contacts.

On arrival at the ED, the patient’s vital signs were as follows: blood pressure 115/77 mmHg, heart rate 82 beats per minute, respiratory rate 24 per minute, temperature 38.8°C, and oxygen saturation 92% on 3L of oxygen via nasal cannula. Neurologic assessment is consistent with profound impairment with no observable responses to painful stimuli. Her physical examination revealed complete left-sided flaccidity involving the arm and leg and non-purposeful movements on the right side. Additionally, her pupils were bilaterally reactive but sluggish. She was given naloxone (400 mcg) and flumazenil (0.5 mg) for possible substance overdose. 

**Figure 1:**
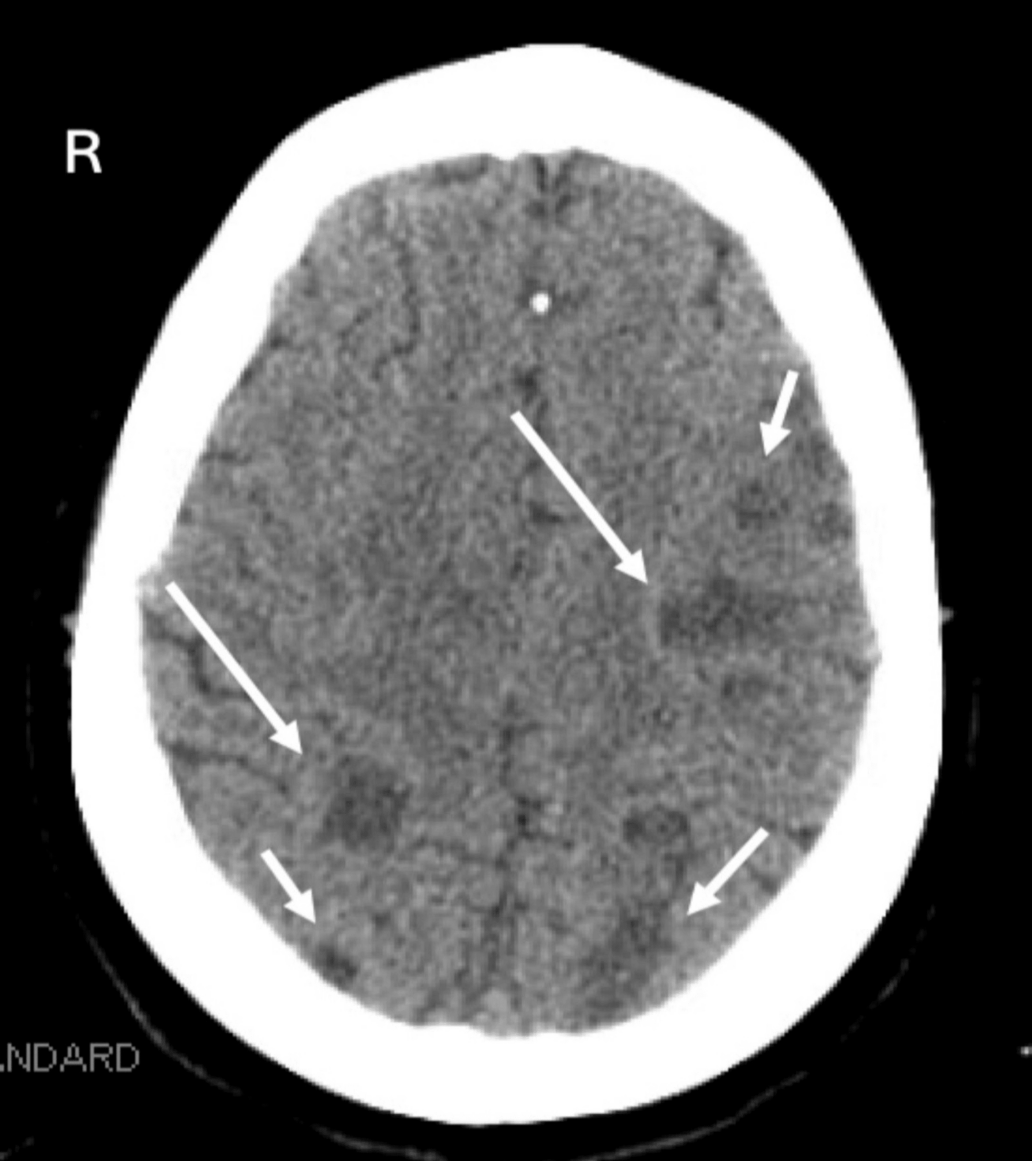
Non-contrast CT of the head showing multiple low-attenuation lesions scattered throughout both cerebral hemispheres (white arrows) and in the periphery of the right cerebellar hemisphere, consistent with acute ischemic changes.

**Figure 2:**
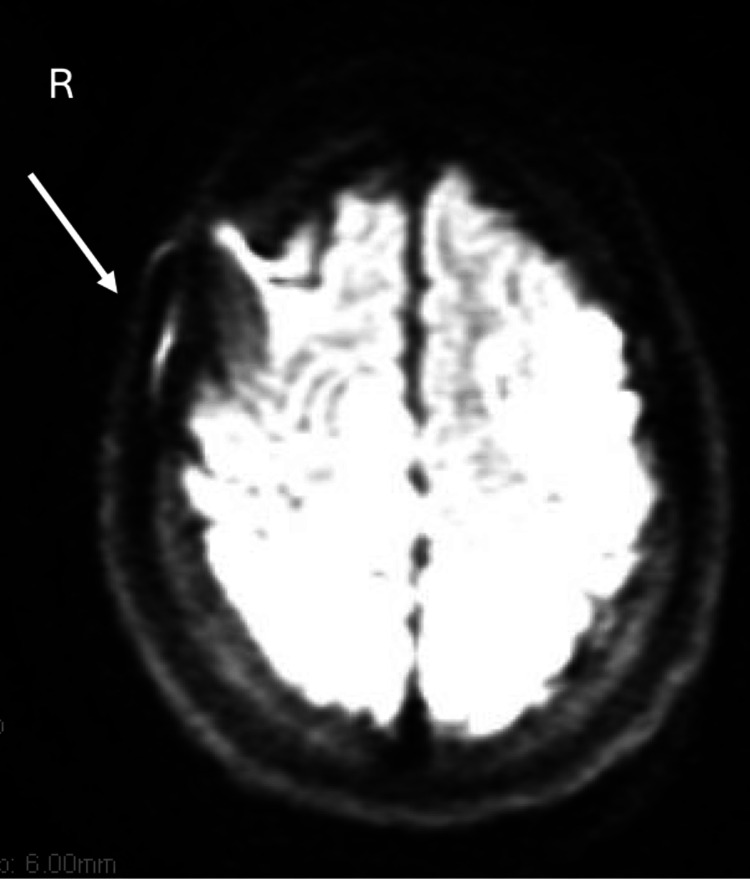
MRI of the brain demonstrating an area of acute ischemic injury (arrow) as one of several sites resulting from vascular compromise. Image quality is limited due to motion artifact.

Emergent non-contrast computed tomography (CT) of the brain demonstrated multiple lesions scattered across both hemispheres with mild mass effect (Figure 1). Subsequent magnetic resonance imaging (MRI) of the brain, performed with and without contrast, revealed acute ischemic injuries across multiple vascular territories and significant vascular compromise of the left carotid artery (Figure 2). Electrocardiogram showed sinus rhythm, and chest radiography did not reveal acute abnormalities. These findings prompted admission to the medical intensive care unit (MICU) for further evaluation and management. 

Approximately 30 minutes after admission into the MICU, she became more arousable and was able to respond to simple questions. She was alert and oriented to person, place, and time, and she denied back pain. Neurological examination revealed left-sided hemiplegia and right-sided Horner's syndrome characterized by anhidrosis and miosis, with preserved facial symmetry. Pulses were palpable bilaterally in the femoral arteries and dorsalis pedis and confirmed with bedside Doppler assessment.

Laboratory investigations (Table 1) revealed an elevated white blood cell count (32.98x10^3^/µL), combined anion-gap and non-anion gap metabolic acidosis (arterial pH 7.34, anion gap 18, bicarbonate 10.4 mmol/L, lactic acid of 7.5 mmol/L), acute kidney injury (creatinine 2.3 mg/dL), rhabdomyolysis (creatinine kinase 1364 U/L), and acute liver injury (aspartate aminotransferase (AST) 3,902 U/L, alanine aminotransferase (ALT) 1,234 U/L). Ammonia levels were elevated at 122 µmol/L. 

**Table 1:** Laboratory test results Asterisk indicates abnormal level AST: aspartate aminotransferase; ALT: alanine aminotransferase

Laboratory Test	Patient Values	Reference Range
White blood cell count (×10³/μL)	32.98*	4.0–11.0
Arterial pH	7.34*	7.35–7.45
Anion gap (mmol/L)	18*	8–16
Bicarbonate (mmol/L)	10.4*	22–28
Lactic acid (mmol/L)	7.5*	0.5–2.2
Creatinine (mg/dL)	2.3*	0.6–1.3
Creatine kinase (U/L)	1,364*	22–198
AST (U/L)	3,902*	10–40
ALT (U/L)	1,234*	7–56
Ammonia (μmol/L)	122*	15–45

Given the patient’s initial presentation with acute confusion, fever, left-sided weakness, and recent methamphetamine use, the leading differential diagnoses included toxic-metabolic encephalopathy, acute ischemic stroke, and infective endocarditis with possible septic emboli. Her multiorgan dysfunction, elevated inflammatory markers, and scattered brain lesions further supported concern for systemic infection or drug-related vasculopathy. These considerations prompted empiric treatment with naloxone, flumazenil, and broad-spectrum antibiotics, as well as further diagnostic imaging.

**Figure 3:**
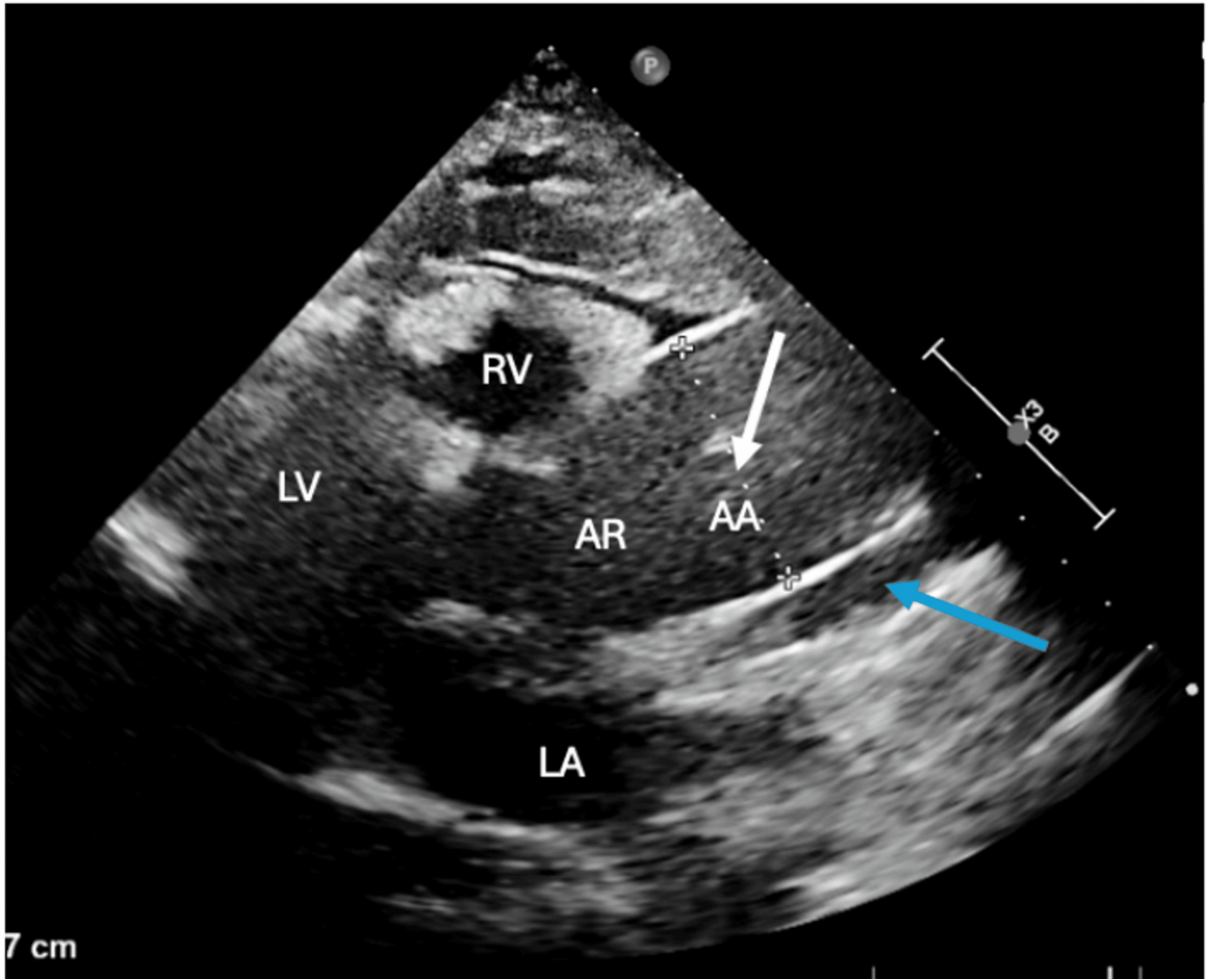
TTE in the parasternal long axis view exhibiting the Stanford type A aortic dissection, with dilatation of the ascending aorta (measuring 4.5 cm). The white arrow points to the true lumen, and blue arrow points to the false lumen. LA: left atrium; LV: left ventricle; RV: right ventricle; AR: aortic root; AA: ascending aorta; TTE: transthoracic echocardiography

Bedside transthoracic echocardiography (TTE) was performed to evaluate for infective endocarditis or structural cardiac abnormalities. Approximately two hours into admission, TTE revealed an aortic root dilation with a torn intimal flap and a small pericardial effusion, raising suspicion for aortic dissection (Figure 3). A trileaflet aortic valve was visualized with no signs of stenosis. 

**Figure 4:**
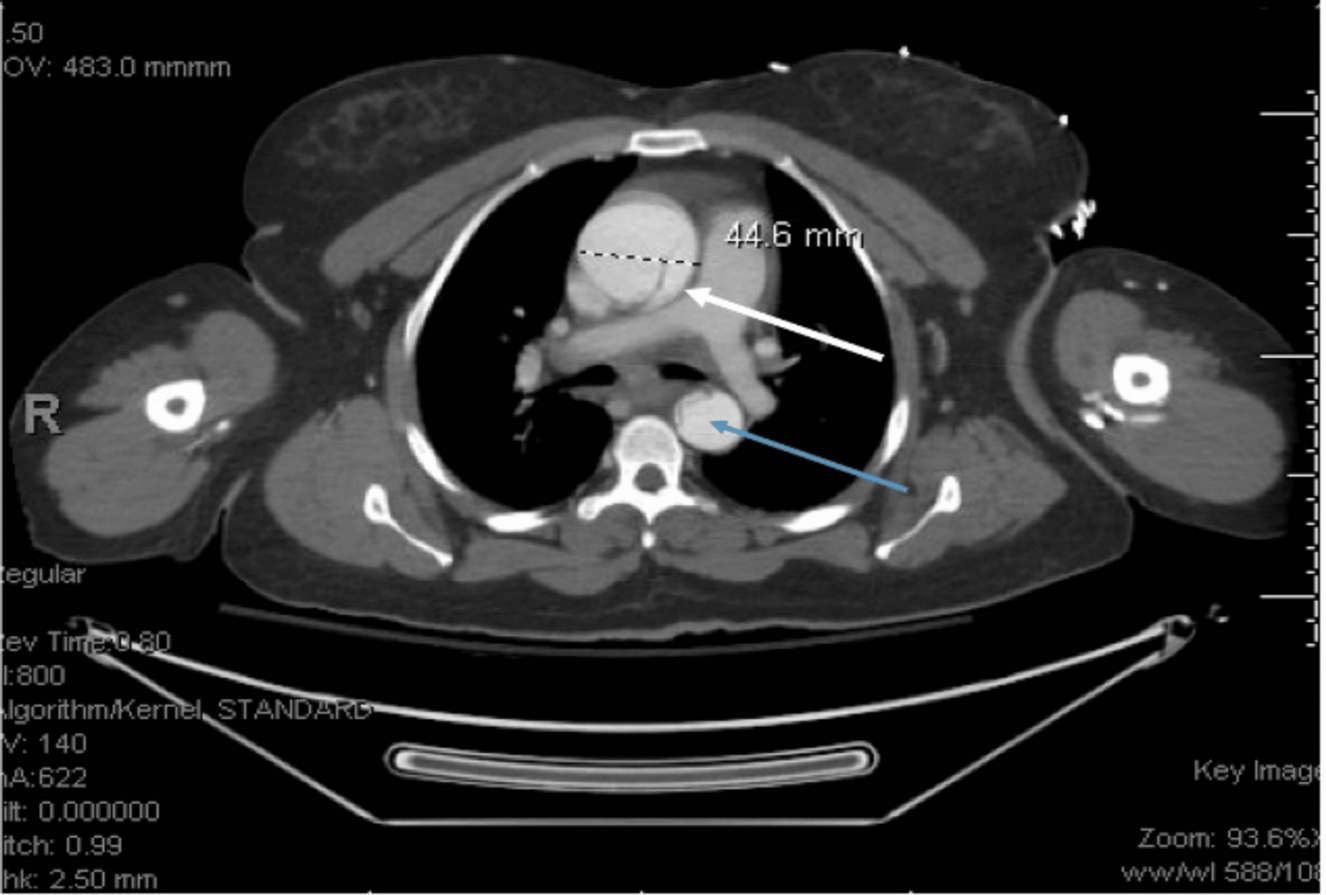
CT angiography of the chest, abdomen, and pelvis illustrating a Stanford Type A aortic dissection. The dissection flap extends into the brachiocephalic artery and proximal right common carotid artery, with partial occlusion of the proximal left common carotid artery. The ascending aorta is enlarged, measuring 4.5 cm in diameter (white arrow). The smaller more densely opacified true lumen is compressed anteriorly along the length of the descending thoracic aorta (blue arrow).

**Figure 5:**
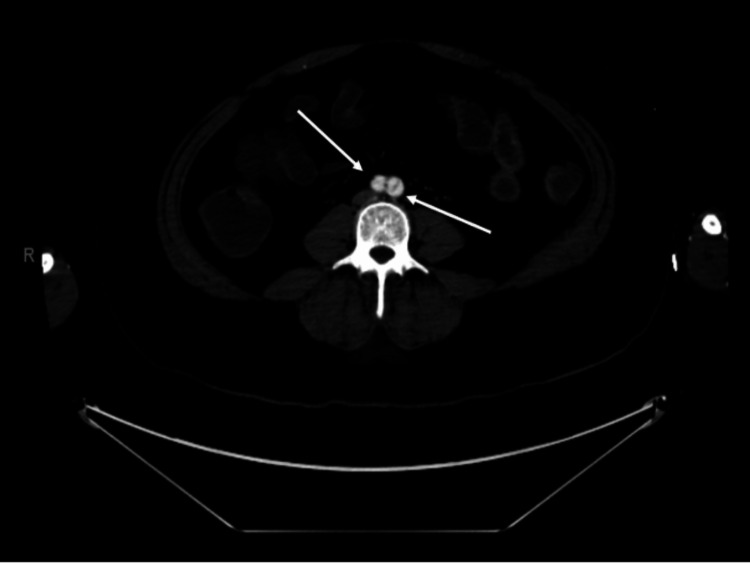
CT angiography showing the Stanford Type A dissection flap extending through the common iliac bifurcation into the left common iliac artery and its branches, including the left external and internal iliac arteries (white arrows).

**Figure 6:**
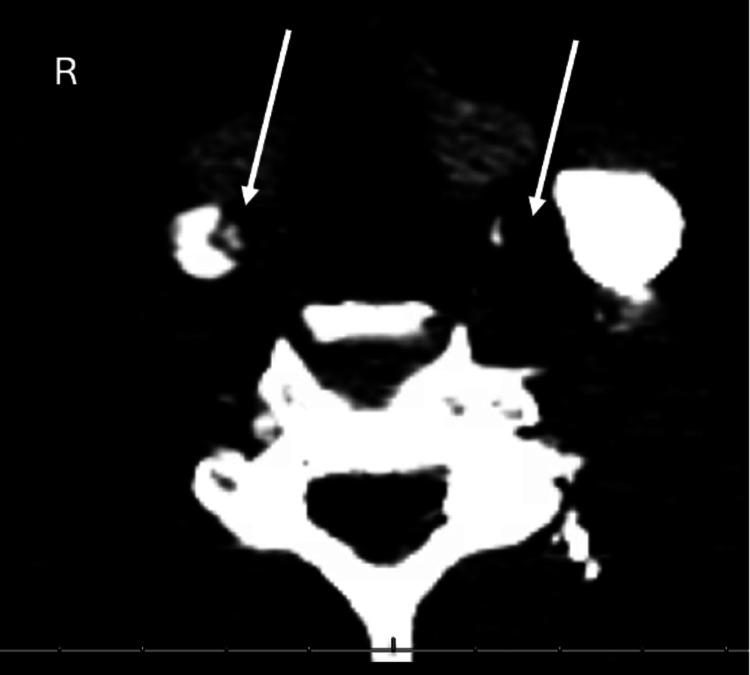
CT angiography of the head and neck revealing the Type A aortic dissection extending into the bilateral common and internal carotid arteries, with near-total occlusion of the left internal carotid artery (white arrows). Image quality is limited due to motion artifact.

At this time, the patient had roughly equal blood pressure measurements in the bilateral upper extremities. Emergent CT angiography (CTA) of the chest, abdomen, and pelvis showed a 4.5 cm ascending aortic dilation with a smaller, densely opacified true lumen compressed anteriorly along the descending thoracic aorta (Figure 4). The celiac axis and inferior mesenteric artery were small in diameter but originated from the true lumen and showed good contrast enhancement. The right renal arteries appeared to arise from the smaller true lumen, but the left renal artery originated from the false lumen. The dissection extended into the iliac arteries bilaterally (Figure 5). CTA of the head and neck showed Stanford Type A aortic dissection extending into the bilateral common and internal carotid arteries, with near occlusion of the left carotid artery (Figure 6). These findings confirmed the diagnosis of Stanford Type A aortic dissection.

The patient was initiated on an intravenous (IV) labetalol infusion (5 mg/mL) to maintain systolic blood pressure between 100-120 mmHg per guidelines [1], IV morphine (2 mg every three hours as needed) for pain control, vancomycin (1000 mg in normal saline) and piperacillin/tazobactam (3.375 g in normal saline) for sepsis (source control pending), and sodium bicarbonate (150 mEq/1,000 mL in dextrose 5% infusion) drip to treat the severe combined anion gap and non-anion gap metabolic acidosis. 

Vascular surgery, cardiothoracic surgery, and interventional neurology teams were consulted for emergent surgical intervention. However, given her delayed presentation, extensive dissection, and poor prognosis, surgical intervention was deemed futile. The patient was managed conservatively and succumbed to her condition on the second day of admission.

The patient’s young age and absence of any clear predisposing conditions raised the possibility of an underlying genetic aortopathy. This suspicion was further supported by her distinct phenotypic features observed by admitting clinicians, including a small chin and thin nose, which are characteristic of certain connective tissue disorders. Comprehensive genetic testing for familial aortopathies was performed using next-generation sequencing with deletion and duplication analysis of known associated genes. Genes tested for genetic aortopathy included: *ACTA2*, *BGN*, *CBS*, *COL3A1*, *COL5A1*, *COL5A2*, *EFEMP2*,* FBN1*, *FBN2*, *FLNA*, *LOX*, *MAT2A*, *MED12 *(c.3020A>G (p.Asn1007Ser) variant only), *MFAP5*, *MYH11*, *MYLK*, *NOTCH1*, *PLOD1*, *PRKG1*, *SLC2A10*, *SMAD2*, *SMAD3*, *SMAD4*, *SMAD6*, *TGFB2*, *TGFB3*, *TGFBR1*, and *TGFBR2*. All results were negative.

lthough further investigations with whole-exome sequencing was discussed to find out potential underlying etiologies, it was not pursued due to resource limitations at the time. The etiology of her dissection, therefore, remains uncertain, highlighting the need for continued investigation into non-traditional or idiopathic causes of aortic dissection in younger patients.

## Discussion

A Stanford Type A aortic dissection presenting primarily with neurological symptoms is a rare and diagnostically challenging scenario, particularly when unaccompanied by the hallmark symptom of chest or back pain. In this case, the red flags for aortic dissection included left-sided hemiparesis and the development of right-sided Horner's syndrome. These symptoms were attributable to dissection-related occlusion of the left internal carotid artery. The pathophysiological connection between aortic dissection and Horner’s syndrome relies on the anatomical proximity of the aortic arch to the sympathetic chain. The interruption of the oculosympathetic pathway leads to the classic signs of Horner’s syndrome on the affected side [4]. In the current case, the patient’s rapid improvement in mental status (approximately 30 minutes after admission into the MICU) likely reflected transient arterial occlusion during the acute propagation phase of the dissection.

Once bedside TTE raised suspicion for a Type A dissection by revealing aortic root dilation and a torn intimal flap, confirmatory CTA of the chest, abdomen, and pelvis was performed to localize the intimal tears. The resulting diagnostic clarity facilitated targeted management decisions. Unfortunately, the delayed presentation and extensive nature of the dissection precluded surgical intervention in this case, highlighting the importance of early recognition and imaging in improving outcomes for this life-threatening condition.

Although aortic dissections with neurological symptoms have been reported, they predominantly occur in patients over 60 years of age, often with established risk factors such as chronic hypertension, and are frequently accompanied by the absence of one or more peripheral pulses [5]. In contrast, our patient’s presentation was unusual in several key respects. She developed a Stanford Type A aortic dissection in her 30s, significantly younger than the typical mean age of 52 years [6]. Moreover, she lacked traditional risk factors such as chronic hypertension, connective tissue disorders, atherosclerosis, known aortic aneurysms, or a family history of aortic disease [7]. Aortic dissection is especially rare in younger women [8], further underscoring the atypical nature of this case. Peripheral pulses were intact bilaterally, confirmed by Doppler, making the diagnosis even more unexpected.

Methamphetamine use has been implicated as a potential risk factor for aortic dissection [9]. Aortic dissection typically results from an intimal tear secondary to degeneration of the aortic media. Methamphetamine use can precipitate this process by inducing sudden and severe hypertensive surges, which increase shear stress on the aortic wall. These episodes, combined with methamphetamine’s potent vasoconstrictive properties, can accelerate medial degeneration and compromise vascular integrity [10]. In our patient, negative findings from extensive genetic testing and the absence of other systemic pathology support methamphetamine use as the likely etiology. Whole-exome sequencing was discussed but not pursued due to resource limitations. However, genetic testing does not exclude all aortopathies, as some pathogenic or novel variants may remain undetected.

The absence of pain in aortic dissection, as observed in this patient, may result from the disruption or occlusion of aortic-side branches supplying the brain, spinal cord, or peripheral nerves. One study reported that the absence of abrupt pain lowers the likelihood of aortic dissection, with a negative likelihood ratio of 0.3 (95% CI: 0.2-0.5) [11]. Conversely, the presence of neurological deficits substantially increases the likelihood of aortic dissection, with positive likelihood ratios ranging from 6.6 to 33.0 [11].

The Stanford classification system differentiates Type A from Type B aortic dissections based on whether the ascending aorta is involved. This distinction is crucial in guiding management, as Type A dissections are a surgical emergency, whereas Type B dissections are usually managed medically, focusing on optimizing blood pressure to reduce shear forces on the vessel wall. Aortic dissections may propagate distally, involving the spinal, visceral, and peripheral arteries, further complicating the clinical picture [1].

## Conclusions

This case highlights the importance of maintaining a high index of suspicion for aortic dissection, even in younger patients who present without classic symptoms such as chest or back pain. Neurological findings, including Horner’s syndrome, may be the first or only sign of a life-threatening dissection. Clinicians should recognize that the absence of traditional risk factors does not exclude the diagnosis. In atypical presentations, bedside TTE can raise suspicion, but CTA remains the gold standard for definitive diagnosis. Early imaging and clinical suspicion are essential, as timely recognition and surgical intervention are critical to improving outcomes in Stanford Type A aortic dissections. This case also supports the inclusion of atypical dissection presentations in simulation scenarios and case-based teaching modules to strengthen diagnostic reasoning in acute care and neurology settings.
